# Cholinergic Interneurons Are Differentially Distributed in the Human Striatum

**DOI:** 10.1371/journal.pone.0001174

**Published:** 2007-11-14

**Authors:** Javier Bernácer, Lucía Prensa, José Manuel Giménez-Amaya

**Affiliations:** Departamento de Anatomía, Histología y Neurociencia, Facultad de Medicina, Universidad Autónoma de Madrid, Madrid, Spain; University of Auckland, New Zealand

## Abstract

**Background:**

The striatum (caudate nucleus, CN, and putamen, Put) is a group of subcortical nuclei involved in planning and executing voluntary movements as well as in cognitive processes. Its neuronal composition includes projection neurons, which connect the striatum with other structures, and interneurons, whose main roles are maintaining the striatal organization and the regulation of the projection neurons. The unique electrophysiological and functional properties of the cholinergic interneurons give them a crucial modulating function on the overall striatal response.

**Methodology/Principle Findings:**

This study was carried out using stereological methods to examine the volume and density (cells/mm^3^) of these interneurons, as visualized by choline acetyltransferase (ChAT) immunoreactivity, in the following territories of the CN and Put of nine normal human brains: 1) precommissural head; 2) postcommissural head; 3) body; 4) gyrus and 5) tail of the CN; 6) precommissural and 7) postcommissural Put. The distribution of ChAT interneurons was analyzed with respect to the topographical, functional and chemical territories of the dorsal striatum. The CN was more densely populated by cholinergic neurons than the Put, and their density increased along the anteroposterior axis of the striatum with the CN body having the highest neuronal density. The associative territory of the dorsal striatum was by far the most densely populated. The striosomes of the CN precommissural head and the postcommissural Put contained the greatest number of ChAT-ir interneurons. The intrastriosomal ChAT-ir neurons were abundant on the periphery of the striosomes throughout the striatum.

**Conclusions/Significance:**

All these data reveal that cholinergic interneurons are differentially distributed in the distinct topographical and functional territories of the human dorsal striatum, as well as in its chemical compartments. This heterogeneity may indicate that the posterior aspects of the CN require a special integration of information by interneurons. Interestingly, these striatal regions have been very much left out in functional studies.

## Introduction

The striatum plays a key role in motor, cognitive and motivational processes [1], [2]. The projection neurons of this structure are a subset of γ-aminobutyric acid (GABA)-containing medium-sized spiny cells [3] that are strongly influenced by local circuit neurons (Golgi type II cells) [3], [4]. Striatal interneurons are grouped in two broad categories: giant aspiny interneurons (aspiny II cells of DiFiglia et al., 1976), and medium-sized interneurons (aspiny I and III cells of DiFiglia et al., 1976). The projection neurons account for 96% of the striatal cells in rodents [5], but, in humans, this percentage decreases to 74% while the number of local circuit neurons increases significantly [6].

The giant aspiny striatal interneurons are cholinergic neurons, which provide the main source of acetylcholine (ACh) to the striatum. Although few in number (1–2% of the total cell population of striatum) [7], the cholinergic interneurons are among the largest striatal cells and have extremely dense axonal arbors. These cells receive prominent synaptic contacts from the substantia nigra [8]–[10], thalamus and cortex [11], [12], and modulate the activity of the striatal projection neurons [13]–[16] and GABAergic interneurons [4], [17]. In the striatum, dopamine inhibits ACh release from the cholinergic interneurons *in vivo* and *in vitro* [10], [18]–[22]. Furthermore, cholinergic neurotransmission in the rat striatum is also modulated by the neuropeptide somatostatin [23], [24], which is released by a subset of medium-sized GABAergic interneurons [25]. The cholinergic interneurons of the striatum are involved in the processing of motivationally relevant events [26], synaptic plasticity [14], [27], and learning [28], and their number is decreased in certain neurodegenerative and psychiatric diseases [29]–[32].

Cholinergic neurons are widely distributed in the human striatum and populate the two major chemical compartments, matrix and striosomes [33]–[35], and particularly the periphery of the latter [36]. However, whether the density of this cell population varies in the different topographical and functional territories of the dorsal striatum is still unknown. Nor is it known, either, whether the compartmental organization of these neurons is alike in every striatal sector. The present study aims at clarifying these questions since the answers should help us to reach a deeper understanding of the cellular composition of the normal striatum, and of the effects of neurodegenerative and psychiatric disorders in this context. Some of the results reported here have been published in abstract form elsewhere [37].

## Materials and Methods

### Tissue preparation

The biological samples of postmortem human brain material used in the present study were obtained from nine adult individuals (7 males and 2 females) of different ages without clinical or pathological evidence of neurological or psychiatric disorders (Table 1), and were provided by the Banco de Tejidos Neurológicos de Navarra (Clínica Universitaria and CIB), the Departamento de Anatomía Patológica (Clínica Universitaria, Universidad de Navarra, Pamplona) and Hospital Ramón y Cajal (Madrid). At the time of the decease, the relatives of the patients were asked for authorization to perform the medical autopsy. Then, many medical samples were anonymized and kept in the hospital for research purposes. The biological samples of the present study were provided by these Departments after the approval of our specific project by the corresponding Ethical Committees of the hospitals where the samples were taken (Clínica Universitaria and Universidad Autónoma de Madrid). All the cases used in this study were obtained between 2001 and 2004.

**Table 1:** Clinical data on the human cases used in this study

Case	Sex	Age (Years)	Postmortem delay (h)	Weight* (g)	Cause of death	Use§
1	Male	35	4	1250	Cardiac arrest	1,2,3
2	Female	58	6	1345	Gastrointestinal hemorrhage	1,3
3	Male	66	17	1385	Gastric adenocarcinoma	1,2,3
4	Male	67	4.5	1280	Bilateral pneumonia	1,3
5	Male	74	7	1090	Gastric sarcoma	1,2,3
6	Male	63	12	1441	Prostate carcinoma	1,3
7	Female	20	2	1100	Cystic fibrosis	1,2,3
8	Male	78	6	1200	Lung carcinoma	1,2,3
9	Male	50	6	1250	Pulmonary disease	1,2,3

* Weight of the whole unfixed brain

§ 1, quantification of the volume of ChAT-ir interneurons; 2, estimation of the density of ChAT-ir interneurons; 3, compartmental distribution of the ChAT-ir interneurons

The brains were cut into 0.5 cm-thick slices that were fixed in a solution containing 4% paraformaldehyde in 0.125 M phosphate buffer pH 7.4 (PB) with 0.2% picric acid at 4°C for ten days. The slices were immersed in 15% sucrose in PB at 4°C for at least another seven days before cutting. Those brains that were not sliced immediately were stored in a mixture of 0.1 M PB saline pH 7.4 (PBS) with 15% sucrose and 0.1% sodium azide. Samples were cut along the coronal plane with a freezing microtome into 50 µm-thick coronal sections that were serially collected in a cryoprotective solution containing 0.05M PB (pH 7.4), with 30% ethylene glycol and 30% glycerol.

### Immunohistochemistry procedures

#### Single immunostaining

Series of sections were treated to reveal choline acetyltransferase (ChAT)-immunostaining. We used a polyclonal goat antiserum (Chemicon, Temecula, CA; product number AB144P) prepared against human placental ChAT that was affinity-purified. This antibody stained a single band of 68–70 kD on the Western blot (manufacturer's technical information). All neuronal staining was abolished when 1 ml of the diluted primary antibody (1∶500) was preincubated with 10 µg of ChAT. Once selected, the sections were rinsed in PBS and treated in a solution containing 50% ethanol (1∶3) and 3% H_2_O_2_ (2∶3) for 30 minutes to inactivate endogenous peroxidase activity. After three more rinses in PBS, the slices were incubated in the solution containing the primary anti-ChAT antibody (1∶500 dilution) and the appropriate normal serum (2% normal rabbit serum) for two days. All of the solutions included PBS and 0.1% Triton X-100. After several rinses in PBS, the sections were reincubated for another 90 minutes at room temperature in a solution containing the respective biotinylated secondary antibody (antigoat IgG made in rabbit, 1∶250; Vector Labs, Burlingame, CA). Then, and after several rinses in PBS, the sections were immersed for 90 minutes at room temperature in a 1∶125 avidin-biotin complex solution (ABC, Vector Labs) according to the method of Hsu et al. [38]. The sections were developed by placing them in a medium containing 0.05% 3,3′-diaminobenzidine tetrahydrochloride (DAB, Sigma, St. Louis, MO) and 0.003% H_2_O_2_ (from a 30% commercial solution) in 0.05 M Tris buffer pH 7.6 at room temperature. The reaction was stopped by rinses in Tris buffer. Control sections were incubated omitting either the primary or the secondary antibody to test the affinity of the secondary antibody and the ABC solution.

#### Double immunostaining

In order to analyze the distribution of the ChAT-ir neurons with respect to the matrix/striosome compartments, some sections were chosen at each anteroposterior level of the CN and Put and processed for ChAT (as described above) and enkephalin (ENK). The ENK immunoreactivity stains the striosomes, specially their peripheral region, more intensely than the matrix, and the resulting stain is suitable for precisely outlining striosomal boundaries. We used a monoclonal anti-ENK antibody made in mouse (1∶50 dilution, Medicorp, Montreal, Canada; product number 1018, cell line NOC1). This monoclonal antibody is secreted by a hybridoma formed by the fusion of a NSO/1 mouse myeloma cell with a spleen cell from a BALB/C mouse immunized against Leu^5^-ENK conjugated to bovine serum albumin. This antibody does not distinguish between Met^5^-ENK and Leu^5^-ENK in immunohistochemistry. It displays about 40% cross-reactivity with C-Terminal extended Met-ENK hexapeptides and 7% cross-reactivity with the extended heptapeptide (-Arg-Phe-OH), but does not recognize other endogenous peptides. In immunohistochemistry, the antibody recognizes all well established ENK-ir sites but does not bind to areas known to contain β-endorphin or dynorphin (manufacturer's technical information). All striosomal staining was abolished when 1 ml of the diluted primary antibody (1∶50) was preincubated with a mixture of 30 µg of Leu^5^-ENK and 30 µg of Met^5^-ENK. The chosen slices were incubated for two days in a solution containing both primary antibodies before ChAT immunohistochemistry processing and development with DAB solution. After this, the sections were thoroughly rinsed in PBS and incubated in the biotinylated secondary antibody solution for ENK (antimouse IgG made in horse, 1∶250; Vector Labs), and the ABC solution as described above. The development was done in a nickel-DAB solution (0.024% DAB, 0.295% nickel ammonium sulphate and 0.003% H_2_O_2_ from a 30% commercial solution), and was stopped as soon as possible to avoid masking the ChAT labeling of the interneurons. We alternated the order of the incubations and developments, and observed that the ChAT/DAB protocol, followed by the ENK/nickel-DAB process was the most suitable method to obtain clear labeling and minimize background.

### Data analysis

#### Topographical subdivisions of the striatum

**Figure 1:**
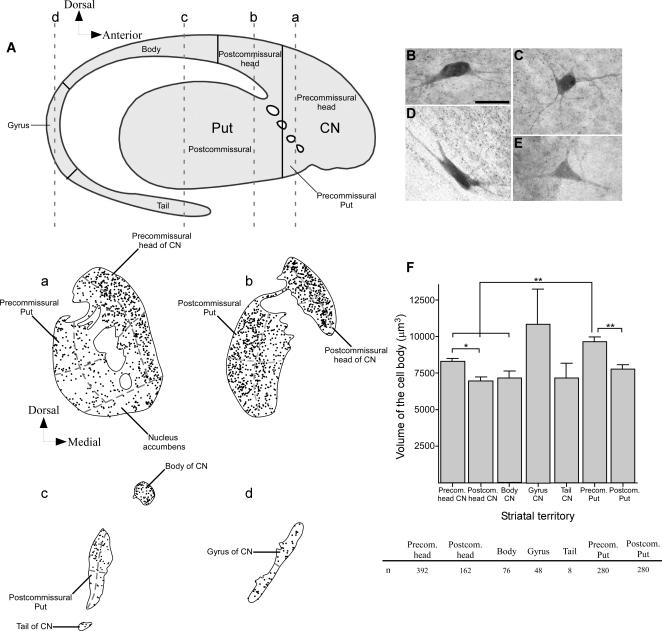
Topographical subdivisions of the human caudate nucleus (CN) and putamen (Put) and general pattern of distribution and volume measurements of striatal cholinergic interneurons. *A*, Drawing of a sagittal view of the striatum illustrating the various territories of the CN and Put examined in this study. Continuous lines indicate the boundaries between adjacent territories. *a–d*, Neurolucida drawings illustrating the distribution of the cholinergic neurons in four coronal sections of the striatum depicted in an anteroposterior order. The anteroposterior levels of these sections are indicated by dashed lines in *A*, and the various sectors in which each striatal territory was subdivided in the present study are depicted by dashed lines in a–d. *B–E*, photomicrographs showing various cholinergic interneurons with an ovoid (*B*), globular (*C*), fusiform (*D*) and triangular (*E*) perikaryon. *F*, bar graph illustrating the mean volume of the cell bodies of the cholinergic cells in the different striatal territories. Significant (*, 0.05>P>0.01) and highly significant (**, P<0.01) differences are indicated. CN, caudate nucleus; n, number of cells analyzed in each striatal region; Put, putamen. Scale bar in B, 40 µm.

The CN and the Put were respectively subdivided into five and two anteroposterior territories (Fig. 1*A* *a–d*) [39]. The anterior commissure was used as a landmark to separate the precommissural and postcommissural striatum. Precommissural striatum includes the *precommissural head* of the CN and the *precommmissural Put* (Fig. 1*A*). The *nucleus accumbens,* which was delimited from the dorsal striatum as described by Selden and colleagues [30], was not included in this study because it shows numerous hodological and compartmental differences with the dorsal striatal tissue and, therefore, other chemical markers are required to visualize its core and shell domains. The postcommissural striatum comprises the following striatal tissue in the CN and the Put. The CN *postcommissural head* includes the CN territory that lies posterior to the anterior commissure up to the level at which the hypothalamic mammillary nuclei disappear. The CN continues posteriorly with the *body*, which ends when it bends ventrally and gives rise to the *gyrus of the CN.* Finally, the *tail of the CN* continues into the temporal lobe. The *gyrus* is, therefore, the most posterior region of the striatum and lies between the body and the tail of the CN. The *postcommissural* territory of the *Put* is larger than its precommissural counterpart (Fig. 1*A*), and includes the posteroventral aspect of the Put, which is considered to be a “limbic-related” region of the striatum [40].

Our analysis of this experimental material employed the following atlases of the human brain: Schaltenbrand and Wahren [41], Mai et al. [42] and Nowinski et al. [43]. The sections were examined using a Nikon SMZ1500 stereomicroscope (Nikon, Melville, NY) and a Nikon Eclipse 80i microscope (Nikon) equipped with a camera lucida and computerized image analysis system with a DXM1200 digital camera (Nikon).

#### Functional subdivisions of the dorsal striatum

The striatum, based on its cortical inputs, contains three major functional territories, namely: associative, sensorimotor and limbic. These functional domains are largely segregated throughout the striatum [1], [2], [44]. The associative territory almost comprises the whole extension of the CN, with the exception of the dorsolateral rim of its head and a small medial portion of the CN tail, and the precommissural Put. The sensorimotor domain includes the dorsolateral aspect of the CN head, part of the dorsal precommissural Put and the entire postcommissural Put. The main component of the limbic striatum is the nucleus accumbens, although there are other regions in the so-called dorsal striatum in which the limbic projections overlap with the associative ones: the ventral sector of both the CN head and precommissural Put and the medial rim of the CN tail. The only region of the dorsal striatum that exclusively contains limbic projections is the posteroventral Put [45].

Our study has analyzed the density of the cholinergic interneurons in the different functional territories of the human dorsal striatum. Thus, we have compared the data of those sectors in which the cortical projections are mainly associative (dorsomedial sector of the CN head, CN body and CN gyrus), sensorimotor (postcommissural Put, excluding its posteroventral aspect) and limbic (posteroventral Put). We have used the criterion of studying the density of cholinergic interneurons in territories that receive only one type of cortical information, in order to minimize the effect of overlapping projections with different cholinergic neuronal densities.

#### Volume of perikarya

We examined a total of 1,246 cholinergic neurons randomly selected with the optical dissector (see below) throughout the striatum of the nine cases included in this study (Table 1), and measured the volume of their perikarya by means of the nucleator. This is a stereological technique that provides the volume of a given structure, no matter its shape, from the output of the microscope. It consists in placing two randomly-oriented planes over the structure of interest, in this case the perikarya of the cells, and then allowing the software to calculate the volume of the cell body by establishing the sites at which these planes intersect the boundaries of the perikarya.

#### Neuronal distribution and cell count

The density of this type of interneuron was calculated by stereological methods in six cases (Table 1), and determined along the complete anteroposterior length of both the CN and Put following a stereological protocol described elsewhere [39]. The sample fraction we chose produced about 50 coronal sections per brain for analysis. Several sections of cases 2, 4 and 6 (Table 1) did not show a proper neuronal immunostaining, so these brains were discarded for the stereological study.

To determine whether the density of the cholinergic neurons varied along the dorsoventral and mediolateral axes in each territory of the CN and Put, we subdivided the largest striatal territories (i.e. the precommissural and postcommissural CN head and the precommissural and postcommisural Put) into four sectors: dorsomedial, ventromedial, dorsolateral and ventrolateral (Fig. 1*A* *a–c*). We also subdivided the gyrus into dorsal and ventral sectors (Fig. 1*A* *d*). Then, we determined the neuronal density in each of these sectors of the different striatal territories.

Cholinergic interneuronal density was analyzed using the optical dissector, an unbiased stereological method [46]–[48], as described previously by Martin and colleagues [49]. The area of the striatum to be analyzed was selected at 4x and the neurons were counted at 20x magnification, using an Olympus microscope (Olympus Optical Co. Europe GmbH, Hamburg, Germany). This microscope was connected to a JVC TK-C1380 video camera (JVC Spain, Barcelona, Spain) and supplied with a motorized stage connected to a Dell OptiPlex computer. We used the CAST package software (Visiopharm, Hørsholm, Denmark) to command the movement of the motorized stage along the XY axes and to provide an automatic selection of microscopic fields, which were then captured by the video camera and projected onto the monitor. This same software generates the dissector grid that was superimposed over the microscopic field projected onto the monitor.

The volume of the dissector (V_dis_) was calculated by multiplying the area of the dissector grid (29,285 µm^2^) by the distance between the two focal planes, which were measured with a microcator (Heidenhain, Traureut, Germany) connected to the Z axis of the microscope stage. The mean thickness of the sections was about 9.2±0.11 µm (mean±standard error of the mean, SEM). The meander sampling was done with the same fraction (3.25%) in every sector of each striatal territory analyzed. The use of this fraction let us analyze a maximum of 100 dissectors in the widest sectors (for example, in any of the four quadrants in which the precommissural and postcommissural head of the CN were subdivided) and one dissector in the smallest (the tail of the CN). The sum of the number of neurons contained in each dissector corresponded to the ΣQ_d_^−^ parameter, and neuronal density (N_v_) was calculated with the formula N_v_ = ΣQ_d_^−^/ΣV_dis _(cells/mm^3^).

The distribution of the cholinergic neurons in various coronal sections of the striatum was drawn with the Neurolucida program (MicroBrightField, Colchester, VT, USA), attached to a Zeiss microscope (Zeiss, Göttingen, Germany).

#### Statistics

We estimated the mean±SEM, the normality and the homogeneity of variances with the values of neuronal densities and volume obtained in the various striatal territories. Since the variances of the samples were rather heterogeneous, the statistical differences in the distribution pattern and volume of these interneurons were calculated using the Kruskal-Wallis and ANOVA tests with post-hoc Tamhane procedure for multiple comparisons. The Mann-Whitney or the *t*-test was used to compare two independent samples. Significant or highly significant differences were respectively considered as 0.05>P>0.01 or P<0.01.

#### Compartmental distribution of the ChAT-ir neurons

This study was performed in all the brains included in this study (Table 1) and used the doubled-labelled ChAT/ENK coronal sections. In all cases, the striosomes were either stained homogeneously for ENK or contained a poorly stained center surrounded by an ENK-rich periphery. The location of the cholinergic neurons within the striosomes and surrounding matrix was drawn at 5x or 10x using a camera lucida. The drawings were scanned and rendered with Canvas (Deneba Systems Inc, Miami, FL) and Adobe Photoshop (Adobe Systems Inc, San Jose, CA) software.

## Results

### Volume of the perikaryon of ChAT-ir striatal interneurons in the CN and Put

ChAT-ir interneurons are widely scattered throughout the CN and Put (Fig. 1*A* *a–d*). These cells display a triangular, polygonal, ovoid, or fusiform perikaryon and numerous aspiny primary dendrites that arborize extensively in the proximity of the cell body (Fig. 1*B*–*E*). The volume of the perikaryon of this neuronal subset varies significantly over the anteroposterior spread of the CN and Put, with cholinergic neurons in the CN gyrus being the most voluminous in the entire striatum (Fig. 1*F*). The ChAT-ir neurons are larger in the precommissural territory of the CN head than postcommissurally, and this difference is statistically significant (ANOVA and Tamhane; P = 0.020; Fig. 1*F*). Likewise, the cholinergic interneurons of the precommissural Put are larger than those located more posteriorly and, statistically, this difference is highly significant (P = 0.001; Fig. 1*F*). After the CN gyrus, the precommissural Put is the striatal territory with the largest cholinergic cells (Fig. 1*F*). Statistical analysis reveals highly significant differences in cholinergic neuron volume between the precommissural Put and either the precommissural and postcommissural head (P<0.001 in both cases) or the body of the CN (P = 0.001; Fig. 1*F*). Age and postmortem delay did not affect the volume of the cholinergic interneurons, as was demonstrated by the linear regression test.

### Distribution of the cholinergic interneurons along the anteroposterior axis of the CN and Put

**Figure 2:**
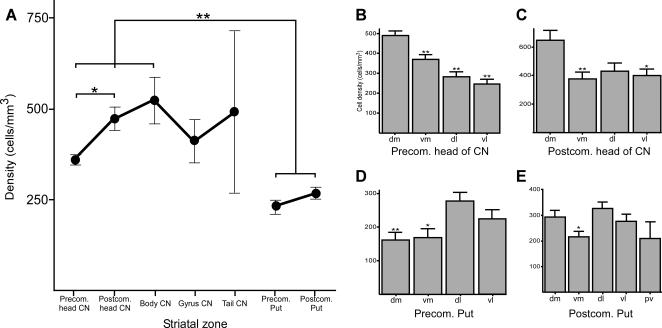
Variation in the density of the cholinergic interneurons throughout the striatum. *A*, line graph showing the mean density of the cholinergic neurons (cells/mm^3^) in the various striatal territories of the CN and Put. *B–E*, bar graphs illustrating the mean density of cholinergic cells in the various sectors (i.e. dm, vm, dl, vl) of the following striatal territories: CN precommissural head (*B*), CN postcommissural head (*C*), precommissural Put (*D*) and postcommissural Put (*E*). Significant (*, 0.05>P>0.01) and highly significant (**, P<0.01) differences are indicated. dl, dorsolateral; dm, dorsomedial; pv, posteroventral; vl, ventrolateral; vm, ventromedial.

**Figure 3:**
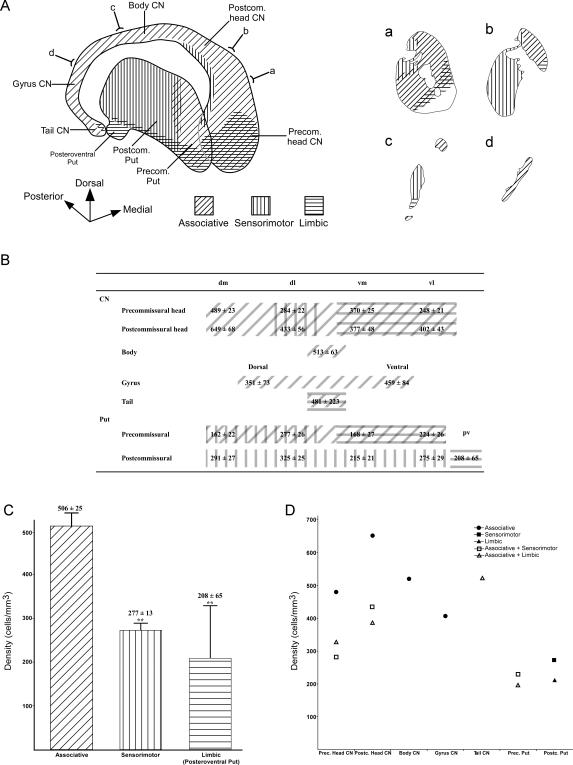
Neuronal density of the cholinergic interneurons in the different functional territories of the human CN and Put. *A*, tridimensional schema showing the associative, sensorimotor and limbic territories of the CN and Put [1], [2]. *a*–*d*, drawings illustrating the functional striatal territories in the coronal plane. The anteroposterior level of each coronal drawing is indicated in the tridimensional schema of the striatum. *B*, table indicating the functional territory represented in each topographical region in which the striatum was subdivided. The functional domains overlap in some sectors of the CN head, in the CN tail and in the precommissural Put. *C*, bar graph showing the mean density of cholinergic interneurons (mean±SEM) estimated in the associative, sensorimotor and limbic territories of the dorsal striatum. Please note that the data shown in this bar graph were obtained by analyzing only those sectors of the striatum that were exclusively associative, sensorimotor or limbic, as shown in *B*. *D*, graph illustrating the cholinergic neuronal density in the various functional territories that are present in every topographical region of the CN and Put. The legend indicates the single or convergent nature of the functional territories. Highly significant (**, P<0.01) differences are indicated in *C*. CN, caudate nucleus; dl, dorsolateral; dm, dorsomedial; Put, putamen; pv, posteroventral; vl, ventrolateral; vm, ventromedial.

The ChAT-ir interneurons populate the anteroposterior extent of the CN and Put (Fig. 1*A* *a–d*). The density of this neuronal subset is about 1.8 times higher in the CN than in the Put and this difference is highly significant (Mann-Whitney U; P<0.001; Fig. 2*A*, 3*B*).

#### CN

The density of cholinergic neurons increases markedly from the precommissural to the postcommissural territories of its head, and continues to increase up to the body, which has the highest ChAT-ir neuronal density of the whole striatum (Figs. 2*A* *,* 3*B*). The tail of the CN, which occurs approximately at the same anteroposterior level as the CN body (see Fig. 1*A*), is also dense in ChAT-ir neurons (Figs. 2*A*, 3*B*). In contrast, in the CN gyrus, a territory that is between the body and the tail, the density of this type of neuron decreases markedly (Figs. 2*A*, 3*B*). The precommissural head is the CN territory with the lowest ChAT-ir cell density (Figs. 2*A*, 3*B*). Statistical analysis reveals significant differences in the density of ChAT-ir neurons between the precommissural and postcommissural CN head (ANOVA and Tamhane; P = 0.034; Fig. 2*A*).

#### Put

The number of ChAT-ir neurons increases from the precommissural to the postcommissural territories of the Put (Figs. 2*A*, 3*B*). This increase is less pronounced than that found between the two divisions of the CN head, and does not reach a statistically significant level (ANOVA and Tamhane; P = 0.942; Fig. 2*A*).

The mean neuronal density of cholinergic interneurons in the human striatum (N_v_) is 361 cells/mm^3^. Although the striatal volume varies according to gender and age, the mean reference volume (V_ref_) for the human striatum may be considered 6.3×10^4^ mm^3^ according to a recent report [50]. Therefore, the total number of cholinergic interneurons in the human striatum may be calculated as N_v_×V_ref_, that is, 2.27×10^7^ neurons.

#### Comparison of ChAT-ir neuronal density between the CN and Put

The variations in the density of cholinergic neurons are highly significant statistically when both the precommissural and postcommissural Put are individually compared to the precommissural head (P<0.001 and P = 0.001, respectively), the postcommissural head (P<0.001 in both cases) or the body of the CN (P<0.001 and P = 0.005, respectively, ANOVA and Tamhane; Fig. 2*A*). However, the difference in density between the global postcommissural CN (the mean density of all the postcommissural territories of the CN) and the postcommissural Put are only significant but not highly so (Mann-Whitney U; P = 0.024).

### Distribution of the cholinergic interneurons along the dorsoventral and mediolateral axes of the CN and Put

To determine whether the distribution pattern of this set of interneurons in each striatal territory shows some variation along their dorsoventral and mediolateral axes, we compared the values of the cellular densities obtained in the various quadrants defined for each of these striatal territories (Figs. 1*A* *a*–*d*, 3*B*).

#### CN precommissural head

The highest density occurs in the dorsomedial quadrant of this territory, followed by the ventromedial, dorsolateral and ventrolateral quadrants in that order (Figs. 2*B*, 3*B*). The difference in density between the dorsomedial aspect of this territory and the other three quadrants is highly significant statistically (ANOVA and Tamhane; P<0.001; Fig. 2*B*).

The number of cholinergic neurons follows a uniformly decreasing mediolateral gradient (Figs. 1*A* *a*, 2*B*). The number of these cells also decreases along the dorsoventral axis, and the decrease is more pronounced in the medial aspect of this territory (Figs. 1*A* *a*; 2*B*). Statistical analysis reveals highly significant differences in the density of ChAT-ir neurons between the lateral and medial halves, as well as between the dorsal and ventral halves (Mann-Whitney U; P<0.001).

#### CN postcommissural head

The dorsomedial quadrant is by far more densely populated by cholinergic neurons than the other three (Figs. 1*A* *b*, 2*C*, 3*B*). Furthermore, the dorsomedial CN postcommissural head has the highest cholinergic density of any part of the striatum (Figs. 2*C*, 3*B*). Statistical analysis reveals highly significant differences in the density of ChAT-ir neurons between the dorsomedial and ventromedial quadrants (ANOVA and Tamhane; P = 0.009) and significant differences between the dorsomedial and ventrolateral quadrants (P = 0.018; Fig. 2*C*). The density of interneurons in the CN postcommissural head is higher in the dorsal half than more ventrally and the difference is statistically significant (Mann-Whitney U; P = 0.035; Figs. 2*C*). In the case of the mediolateral plane, no significant differences are found between the medial and the lateral halves of this territory (P = 0.167).

#### CN gyrus

The ventral half is denser in ChAT-ir neurons than the dorsal half (Fig. 3*B*), but the difference between the two halves is not statistically significant (Mann-Whitney U; P = 0.327).

#### Precommissural Put

The dorsolateral quadrant is the densest in cholinergic interneurons, followed by the ventrolateral, ventromedial and dorsomedial quadrants (Figs. 2*D*, 3*B*). The difference in density is statistically highly significant between the dorsolateral and dorsomedial quadrants (ANOVA and Tamhane; P = 0.007; Fig. 2*D*), and significant between the dorsolateral and ventromedial quadrants (P = 0.029; Fig. 2*D*). The lateral half of this territory shows a higher density in this chemospecific type of neuron than the medial half, and this difference is statistically highly significant (Mann-Whitney U; P<0.001; Figs. 2*D*). There are no significant differences in the density of cholinergic neurons along the dorsoventral plane.

#### Postcommissural Put

As in the precommissural Put, the dorsolateral quadrant is the most densely populated territory and shows significant differences with the ventromedial aspect (ANOVA and Tamhane; P = 0.011; Fig. 2*E*). Cholinergic neuron density is the lowest in the posteroventral aspect of this territory and is also one of the lowest in the entire striatum (Figs. 2*E*, 3*B*). Statistical analysis reveals highly significant differences in ChAT-ir neuron density between the dorsal and ventral halves (Mann-Whitney U; P = 0.002) but no significant differences between the medial and lateral halves (Mann-Whitney U; P = 0.087).

### Distribution of the cholinergic interneurons in the functional territories (associative, sensorimotor and limbic) of the dorsal striatum

In order to analyze the presence of the cholinergic interneurons in the different functional domains of the human striatum, we have calculated the neuronal density in those striatal regions that are considered associative, sensorimotor or limbic based on their corticostriatal connections. We have avoided including those striatal regions that are known to receive overlapping inputs from different functional cortices in this part of the study (Fig. 3A). The associative territories included here are the dorsomedial sector of the head of the CN, the CN body and CN gyrus. The mean density calculated in these associative CN sectors was compared with that obtained in both the postcommissural Put (which is a sensorimotor territory) and the posteroventral Put (limbic sector of the dorsal striatum) (Fig. 3*C*).

Interestingly, the associative striatal domain harbors the highest neuronal density (Fig. 3*C*), and the statistical analysis reveals highly significant differences when it is compared with the sensorimotor (ANOVA and Tamhane; P<0.001) and the posteroventral Put, which is a limbic domain (ANOVA and Tamhane; P<0.001). Although the sensorimotor territory has a greater cholinergic neuron density than the limbic territory of the dorsal striatum, no statistical differences are found when these two functional domains are compared to each other (ANOVA and Tamhane; P = 0.673) (Fig. 3*C*).

We have also analyzed ChAT-ir neuronal density in the various functional domains included in each of the seven territories in which the CN and Put were divided, and these results are depicted in Fig. 3*D*. In the precommissural CN head, the associative domain contains by far the highest neuronal density, followed by its ventral territory in which the associative and limbic projections overlap. In the dorsolateral sector of the precommissural CN head, the associative and sensorimotor inputs converge and cholinergic neuron density was the lowest of all the sectors (Fig. 3*D*). In the postcommissural CN head, the associative domain was also by far the most densely populated, followed by the sectors in which associative/sensorimotor and associative/limbic afferents overlap. The CN body and gyrus are exclusively associative, whereas associative and limbic projections overlap in the CN tail [40]. In the precommissural Put, the associative/sensorimotor territory is more densely populated than the associative/limbic territory. Finally, in the postcommissural Put the neuronal density is higher in its sensorimotor than in the limbic territory (posteroventral Put) (Fig. 3*D*).

### Compartmental distribution of ChAT-ir interneurons in the various striatal territories of the CN and Put

**Figure 4:**
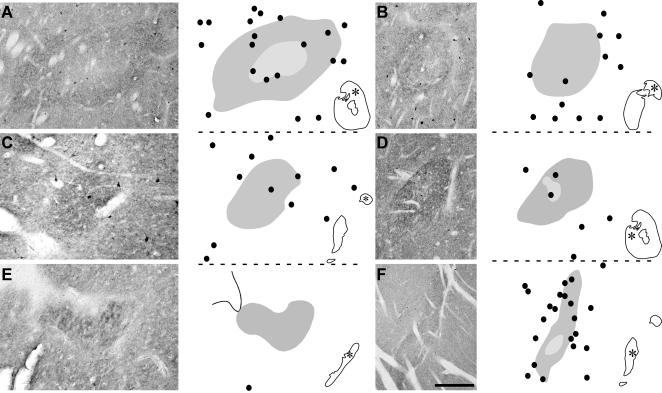
Distribution patterns of the cholinergic interneurons within the striosomes in the various territories of the CN and Put. *A–F*, photomicrographs and camera lucida drawings depicting the distribution of the cholinergic interneurons in six enkephalin-immunoreactive striosomes located in the precommissural head (*A*), postcommissural head (*B*), body (*C*) and gyrus (*E*) of CN, and in the precommissural (*D*) and postcommissural (*F*) Put. The light and dark gray shadings in *A*, *D* and *F* indicate the center and periphery of the striosomes, respectively. The inset on the bottom right of each drawing indicates the location of the depicted striosome. Scale bar, 650 µm (*A*), 1000 µm (*B*), 260 µm (*C*), 1500 µm (*D*), 700 µm (*E*), 1000 µm (*F*).

**Figure 5:**
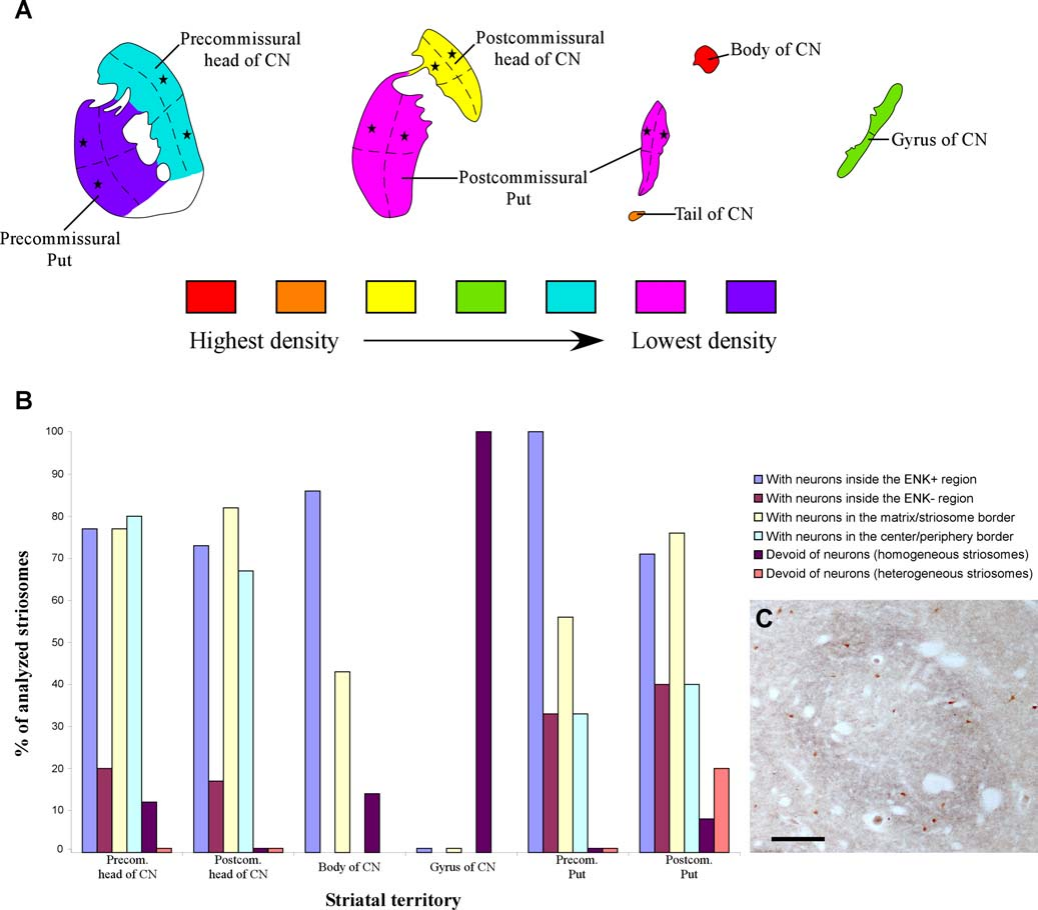
Distribution of ChAT-ir interneurons in the striatum and in its chemical compartments. *A*, gradients of density of cholinergic interneurons in the various territories of the CN and Put. Within each territory, the density of cholinergic neurons is higher in the quadrants indicated by the asterisks. *B*, variation in the distribution of the cholinergic interneurons within the striosomes in the various striatal territories. The bar graph illustrates the percentage of striosomes that fulfil the condition indicated by each color in the legend. *ENK+ region* comprises homogeneous striosomes and the periphery of heterogeneous striosomes. *n* in the X-axis indicates the number of striosomes (homogeneous/heterogeneous) analyzed in each territory. *C*, color photomicrograph taken from a double ENK/ChAT immunostained section showing one striosome and numerous ChAT-ir neurons in the precommissural head of CN. Observe that the periphery of the striosome is more intensely stained for ENK than its center, and that the ChAT-ir neurons occur within the striosome and in the surrounding matrix. CN, caudate nucleus; ENK, enkephalin; Put, putamen. Scale bar, 250 µm (*C*).

The distribution of the cholinergic interneurons with respect to the matrix/striosomes compartments, as visualized with ENK-immunoreactivity was analyzed in each territory of the CN and Put. Immunostaining for this neuropeptide revealed striosomes in every striatal region but the CN tail. In our samples, we could not distinguish between the center and the peripheral region of the ENK-immunostained striosomes located in the body and gyrus of CN. In the rest of the striatum, the striosomes were either uniformly stained for ENK or displayed a center that was poorly stained for ENK surrounded by an ENK-ir-rich periphery (Figs. 4, 5*C*). A total of 35 striosomes were analyzed in the CN (13 in its precommissural head, 11 in its postcommissural head, 7 in its body, and 4 in the gyrus) and 26 in the Put (9 in its precommissural territory and 17 in its postcommissural territory) (Fig. 5*B*).

#### CN

Cholinergic interneurons are present in both the matrix and the striosomes throughout this structure, except in the gyrus. In the precommissural head, many ChAT-ir cell bodies occur within the ENK-ir periphery of the striosomes, and many of these cells are located at the boundaries between either the center and the periphery of the striosomes or the striosome periphery and the extrastriosomal matrix (Figs. 4*A*, 5*B*). This striatal territory contains the highest percentage of striosomes with ChAT-ir neurons in the border between its center and periphery (Fig. 5*B*). Only a few striosomes contain ChAT-ir neurons in their ENK-ir-poor center.

The number of cholinergic interneurons within the striosomes is lower in the postcommissural territories of the CN than in its precommissural head (Fig. 4*A*–*C,E*). This decrease is also evident in the matrix surrounding the striosomes in the CN body and gyrus (Fig. 4*C,E*). In the CN postcommissural head, the intrastriosomal interneurons are confined to the ENK-rich periphery and to the boundaries between either the matrix and the striosomes or the center and periphery of the striosomes (Figs. 4*B*, 5*B*). The striosomes in the CN body are homogeneously stained for ENK and contain none or very few ChAT-ir neurons (Figs. 4*C*, 5*B*). The cholinergic neurons of the CN gyrus are exclusively located in the matrix and at a distance of more than 200 µm from the boundaries of the striosomes (Figs. 4*E*, 5*B*).

#### Put

As in most of the CN, the cholinergic interneurons are present in the matrix and striosomes throughout the Put (Fig. 4*D,F*). The striosomes in the precommissural Put contain notably fewer cholinergic neurons than those in the postcommissural territory of this structure (Fig. 4*D,F*). The intrastriosomal cells are preferentially located in the periphery, and abound at the boundaries between the striosomes and the matrix (Figs. 4*D,F*, 5*B*). Some ChAT-ir neurons are also found at the border between the center and the periphery of the striosomes located in both the pre- and postcommissural territories of the Put (Figs. 4*D,F*, 5*B*).

Regarding the compartmental organization of the cholinergic interneurons with respect to the associative, sensorimotor and limbic territories of the dorsal striatum, our results indicate that there is not a distinctive pattern for each of these functional domains. As we have described above, the location of the cholinergic interneurons with respect to the striosomes in the associative territory of the CN head is rather different from that found in the body or gyrus of CN, which are also associative regions.

## Discussion

The present investigation has demonstrated that, while ChAT-ir interneurons populate the entire striatum, their density, volume and distribution vary through the anteroposterior territory of this structure. The immunohistochemical staining used in this study is considered the most reliable method for identifying cholinergic structures [51], and the ChAT-ir cells in the striatum are undoubtedly interneurons [25], [32], [36], [52]. ENK-immunoreactivity is an excellent stain to visualize the different chemical compartments of the human striatum [36]. The ENK-ir periphery of some striosomes has been referred as an “annular compartment” by several authors [see 33]. However, recent works have demonstrated that this ENK-ir periphery is part of the striosomal compartment [36], [53], and this may be emphasized by the fact that several striosomes are uniformly stained for ENK. We have used double ChAT/ENK immunostaining to identify the striatal compartments and cholinergic neurons in this brain structure. Stereology is a reliable methodology to quantify the number of neurons from measurements made on immunostained microscope sections [47], [49], [54].

Using these techniques, we have observed that the density of these neurons within any given striatal territory varies significantly along the dorsoventral and mediolateral axes. Cholinergic interneurons are significantly more numerous in the CN than in the Put, and their numbers increase from the precommissural to the postcommissural territories of these two striatal components. The CN body contains the highest density of ChAT-ir interneurons, whereas the precommissural Put contains the lowest density. In most striatal territories, the distribution of the ChAT-ir interneurons follows a decreasing dorsoventral gradient. However, the distribution pattern along the mediolateral plane is different in the CN and the Put. Thus, the ChAT-ir cells abound in the medial aspects of the former and in the lateral half of the latter. Concerning the distribution of these neurons in the functional territories of the dorsal striatum, we have found that their density is significantly higher in the associative domain than in the sensorimotor and limbic territories of both the CN and Put.

One important methodological concern when expressing the amount of cholinergic interneurons in terms of their density is brain tissue shrinkage during aging. As shown in Table 1, the weight of the whole unfixed brains did not correlate with the age of the individuals. This observation does not support the existence of significant shrinkage in the older subjects although there is a published report of a moderate loss of volume in the striatum with aging [55].

The main purpose of our study is to demonstrate the selective distribution of the cholinergic interneurons in the human striatum. Since we have used postmortem human brain material from young (20 and 35), middle-aged (50 and 66) and old (74 and 78) individuals (Table 1), the overall data used here to analyze cell distribution were balanced with respect to age. We are aware that if we had calculated the total number of cells in the different regions of the striatum, the possible shrinkage of the tissue would not have affected the results. However, we did not use this method because data gathered from differently-sized striatal territories cannot really be reliable compared.

In addition, our study illustrates that the volume of the perikarya of the cholinergic cells also varies between the different striatal territories. Some studies report a variation in the size of the ChAT-ir interneurons between the dorsal and ventral striatum [51], [56]–[59], as well as between the CN and Put [57]. However, there are no previous reports of a variation in the volume of the ChAT-ir interneurons throughout the whole extent of the CN and Put. Interestingly, our results suggest that the variation in the volume of the ChAT-ir interneurons might be related to the variation in their density. The fact that these interneurons are larger in the striatal territories where there are fewer cells suggests that this increase in size could respond to a need to compensate for the lack of a higher neuronal density.

Regarding the distribution of the ChAT-ir interneurons with respect to the chemical compartmentalization of the striatum, our study demonstrates that: (1) these interneurons populate the matrix, striosomes and the borders between both compartments in every territory of the CN and Put except for the gyrus, where the neurons were found exclusively within the matrix; (2) the amount of cholinergic interneurons inside the striosomes is considerably higher in the CN precommissural and postcommissural heads; and, (3) the CN precommissural head is the striatal territory with the most ChAT-ir interneurons in the border between the ENK-ir-poor center and ENK-ir-rich periphery of the striosomes. Our study also shows that the organization of the cholinergic interneurons with respect to the major compartments does not appear to be related to the type of functional inputs (associative, sensorimotor or limbic) to the striatal territory where these compartments are located.

The CN and Put are considered components of a single neural structure, the striatum, with different functions. Both striatal nuclei are considered to be cytologically identical and composed of multitudinous medium-sized projection neurons and a great diversity of interneurons [25], [60]. However, the relatively large amount of contrasting data on the distribution of the various types of interneurons in the human striatum led us to reconsider this assumption. Previous studies centered on NADPH-diaphorase striatal cells together with the present results have clearly illustrated that there are more interneurons in the CN than in the Put [39]. Similar findings have also been reported regarding the relative numbers of parvalbumin- and calretinin-ir striatal interneurons [61]. Interestingly, it should be noted that the difference in the density of ChAT-ir interneurons between the CN and Put is maintained along the whole anteroposterior axis of the striatum. Therefore, the human CN and Put should no longer be considered cytologically similar structures, at least with respect to their interneuronal density.

Regarding to the cholinergic neuronal density in the functional territories of the human dorsal striatum (i.e. associative, sensorimotor and limbic), the highest density occurs in the associative striatum, and is followed by the sensorimotor and the limbic territories. These findings were expected, since the associative territory is related to more complex information processing, a task that may require a large number of cholinergic interneurons [27]. Given our results, the amount of cholinergic neurons needed for processing the information in the associative, sensorimotor and limbic territories of the dorsal striatum varies significantly, with the density in the associative territory being almost twice as much as in the other territories.

**Figure 6:**
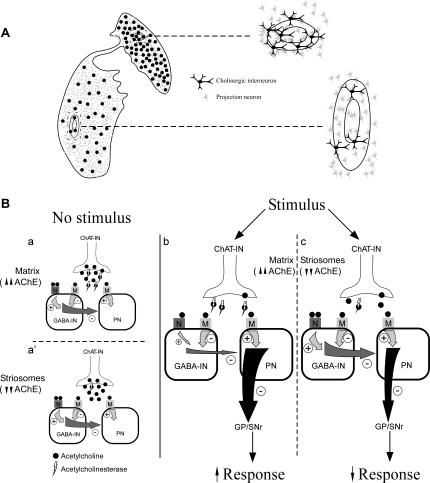
Schematic drawings with functional considerations on the ChAT-ir interneurons. *A*, difference in the density of cholinergic interneurons between the CN and Put. ChAT-ir neurons populate the matrix and the striosomes in the CN and Put but the number of these cells in the two compartments is higher in the former than in the latter. In both striatal components, the cholinergic interneurons abound at the boundaries between the two major compartments and the two striosomal regions. *B*, Variation in the activity of the cholinergic neurons defined as TANs depending on: 1, the presence or absence of a reward-associated stimulus and 2, their location in the matrix or within a striosome. *a–a*′, without stimulus the TANs constantly release ACh, which exerts a dual control on GABAergic interneurons through the nicotinic and muscarinic receptors. Due to the large amount of ACh release by these cholinergic neurons, the greater or lower amount of AChE might not be a limiting factor for the activation of the GABAergic interneuron cholinergic receptors. *b–c*, in the presence of a stimulus the amount of ACh released by the TANs decreases. If ACh is released in the matrix, the high AChE content of this milieu might stop the ACh signal very rapidly at the synaptic cleft, thereby avoiding nicotinic receptor activation on the GABAergic interneurons, and, therefore, facilitating the discharge of projection neurons. If the ACh release is inside a striosome, its low AChE content could facilitate activation of the nicotinic and muscarinic receptors on the GABAergic neurons, which, in turn, would inhibit the projection neurons. ACh, acetylcholine; AChE, acetylcholinesterase; ChAT-IN, cholinergic interneuron; GABA-IN, GABAergic interneuron; GP, globus pallidus; M, muscarinic receptor; N, nicotinic receptor; PN, projection neuron; SNr, substantia nigra *pars reticulata*.

We have also found that these interneurons are more abundant within the striosomes of the CN than in those of the Put (Figure 6*A*). Several reports have described a special activity in the CN during the execution of complex cognitive and motivational tasks [62]–[65]. The performance of these tasks probably requires the integration of the information that is conveyed in the matrix/striosomes compartments, and this would involve the recruitment of a higher number of cholinergic interneurons to communicate the different striatal compartments. The CN and Put each receive a different set of projections from the cerebral cortex and from the thalamus. As many studies in non-human primates have shown, association cortices conspicuously project to the entire anteroposterior extent of the CN as well as to the precommissural Put, whereas more posterior aspects of the Put receive projections from sensorimotor cortices [44], [66]–[70]. With respect to the thalamostriatal system, different thalamic nuclei project selectively to either the CN or the Put [71]–[77]. The different functional activities of the CN and Put in humans have also been demonstrated in a recent fMRI study that has shown how complex tasks activated the CN whereas movement frequency, but not movement complexity, activated the posterior Put [78].

The cholinergic interneurons participate in processing the information that reaches the striatum and is then conveyed to the output structures of the basal ganglia. These structures are targeted by collaterals of corticostriatal and thalamostriatal fibers as well as by dopaminergic inputs from the mesencephalon [10], [25], [79], [80] and, in their turn, they project to striatal projection neurons as well as to gabaergic interneurons [15], [25], some of which express nicotinic receptors [4]. Furthemore, ChAT-ir interneurons are thought to be tonically active neurons (TANs), which participate in the processing of sensory messages reaching the striatum [12], [26], [28]. These cells constantly release ACh and cease their tonic firing after environmental events of reward or aversive motivational significance. The pause in response by the TANs to reward-associated or non-associated stimuli is different depending on their position in the CN or Put [81]. It may be that the result of the momentary TAN inhibition after a given stimulus would vary depending on whether the ACh release by these neurons is in the matrix or the striosomes, since these two compartments have different acetylcholinesterase (AChE) contents. This enzyme, which hydrolyzes ACh into choline and acetic acid, is much more abundant in the matrix than in the striosomes [82]. Although the position of the ChAT-ir cell body does not indicate unequivocally where the ACh is released, the location of cholinergic interneurons within the matrix and the striosomes, as well as in the borders between these compartments, leads to assume that ACh release may happen in both the matrix and the striosomes. In the absence of stimulus, TANs constantly release ACh, which exerts a dual control on GABAergic interneurons through nicotinic and muscarinic receptors [4], [13], [83]. Nicotinic receptor activation requires more ACh than that needed to activate muscarinic receptors [84]. The amount of ACh released by the TANs decreases in the presence of a stimulus [10]. If the ACh is released in the matrix, the high AChE level might rapidly terminate the ACh signal and avoid the activation of the nicotinic receptors. In contrast, if the ACh release were inside a striosome, the low AChE content of this compartment could facilitate ACh activation of the same receptors (Fig. 6*B*). When the nicotinic receptors of the GABAergic interneuron are activated, the interneuron would have a powerful inhibitory effect on the striatal projection neuron. However, if only the muscarinic receptors of the interneuron were activated, the projection neuron would not be inhibited. As a result, the striatal projection neurons would be more strongly suppressed by the GABA interneurons in the striosomes than in the matrix and, therefore, the influence on the output structures would vary considerably between the two compartments depending on their relative amount of ACh (Fig. 6*B*).

Finally, cholinergic interneurons are involved in several pathological processes that affect striatal function, such as Parkinson's disease, Huntington's disease, schizophrenia or progressive supranuclear palsy [14], [29], [32], [85]. Cholinergic interneurons are differentially distributed in the striatum and this distribution may predispose to a more severe affectation of certain striatal regions; this possibility would be an extraordinarily important target for research focused on developing new therapeutic strategies for these diseases.
